# Incidence of cross-border reproductive care and pregnancy outcomes: a retrospective evaluation of IVF conceptions

**DOI:** 10.1007/s10815-025-03449-x

**Published:** 2025-03-22

**Authors:** Suset Rodriguez, Kyara Marquez, Gemma St Louis, Arianna Vazquez, Leonardo Simonelli, Kara Lindsay, Janice Moscoso, George Roshdy Attia

**Affiliations:** 1 University of Miami Miller School of Medicine, Miami, FL USA; 2 New York University Grossman School of Medicine, New York, NY USA

**Keywords:** Cross-border reproductive care, In vitro fertilization, Global health, Reproductive traveling

## Abstract

**Purpose:**

This study aimed to evaluate the incidence of cross-border reproductive care (CBRC) and maternal and neonatal outcomes in pregnancies conceived through in vitro fertilization (IVF), comparing outcomes between pregnancies conceived domestically and through CBRC.

**Methods:**

A retrospective chart review was conducted on 4475 deliveries at a tertiary public hospital from February 1, 2023, to January 31, 2024. Maternal demographics, medical history, delivery outcomes, and complications were compared using statistical tests, with significance set at *p* < 0.05.

**Results:**

Among all deliveries, 95 were conceived via IVF. Among the IVF pregnancies, 23 (24.2%) were conceived through CBRC. While CBRC patients were older on average (40.3 vs. 37.8 years), this difference was not statistically significant (*p* = 0.07). The incidence of hypertensive disorders was significantly higher in CBRC pregnancies compared to domestic IVF pregnancies (69.6% vs. 37.7%, *p* = 0.01). Trends toward increased rates of postpartum hemorrhage (17.4% vs. 5.8%) and multiple gestations (26.1% vs. 11.6%) were also observed in the CBRC group, although these differences did not reach statistical significance. Neonatal outcomes, including rates of preterm birth, fetal growth restriction, and respiratory distress syndrome, were similar between the two groups.

**Conclusion:**

The study highlights a significantly higher percentage of hypertensive disorders among CBRC pregnancies, while other maternal and neonatal outcomes were comparable to domestic IVF pregnancies. These findings underscore the need for further research with larger sample sizes and survey-based studies to better understand patient motivations and clinical risks associated with CBRC.

## Introduction

Infertility affects approximately 1 in 6 adults globally, representing a significant public health concern and a profound emotional, social, and financial burden for those seeking parenthood [1]. Assisted reproductive technology (ART), including in vitro fertilization (IVF), offers hope for many, yet access to these advanced treatments remains restricted and uneven across countries due to disparities in healthcare policies, costs, and legal frameworks. For some, these barriers necessitate cross-border reproductive care (CBRC), where patients travel internationally to obtain fertility treatment unavailable, unaffordable, or restricted in their home countries.

CBRC encompasses a wide range of reproductive medical services, including IVF, donor gametes (eggs or sperm), gestational surrogacy, and, in some cases, abortion services [2]. Patients may pursue CBRC for various reasons: to access higher-quality or broader treatment options, to reduce the financial burden of care, to bypass restrictive legal frameworks, or to seek privacy and cultural comfort. For example, countries with limited availability of donor gametes or prohibitive surrogacy laws often see patients traveling to destinations with more favorable legal and regulatory environments. However, CBRC introduces complexities that extend beyond logistical and financial challenges. Ethical concerns, such as the potential exploitation of women in lower-income countries for surrogacy or gamete donation and health risks associated with varying clinical standards, are significant considerations. Furthermore, patients and offspring may face legal and informational barriers, particularly in navigating issues of parentage, citizenship, and access to donor identities. These complexities highlight systemic inequities in reproductive healthcare access, particularly in high-cost systems like the USA.

Despite CBRC being a growing global phenomenon, its true scope is challenging to measure due to the absence of comprehensive international reporting systems. This gap not only limits our understanding but also impedes efforts to effectively address and manage this issue. Efforts to collect data in destination countries and regions with ART databases have yielded limited results. Recent data from a 2017 study revealed that the percentage of ART cycles conducted for non-US residents increased from 1.2% in 2006 to 2.8% in 2013, with treatments provided to residents from 147 countries [2]. In contrast, the incidence of US residents seeking care abroad is estimated to be lower than that of those coming into the USA. However, despite extensive efforts to gather comprehensive data, a clear and accurate assessment of CBRC among US residents remains unknown.

Understanding the incidence of CBRC among US residents and the associated pregnancy outcomes is critical for evaluating the broader implications of this practice. CBRC introduces complexities such as logistical, financial, and medical challenges that could burden patients. Moreover, it underscores systemic issues within the US healthcare system, including limited affordability and access to ART. This study aims to assess the incidence of pregnancies conceived through IVF performed abroad and to compare pregnancy outcomes between patients undergoing IVF domestically and those seeking CBRC.

## Materials and methods

We conducted a retrospective chart review of delivery records at our institution, an academic tertiary care public facility, from February 1, 2023, to January 31, 2024. The Institutional Review Board of the Miller School of Medicine at the University of Miami approved this study (#20240023). Electronic data for patients with a diagnosis of ART or IVF maintained in our center’s electronic health record system were collected and analyzed. The aim of this review was to identify and assess the incidence of pregnancies conceived through IVF and to evaluate their associated pregnancy outcomes, with a specific focus on CBRC.

Patients were divided into two groups based on where IVF treatment was performed: (1) within the USA and (2) outside of the US. Demographic characteristics, medical history, and delivery outcomes were extracted from medical records. The variables analyzed included maternal age, parity, indication for IVF, gestational age at delivery, mode of delivery, birth plurality, birth weight, and maternal and fetal/neonatal complications. A birth was defined as a vaginal or cesarean birth at or beyond 20 weeks of gestation. Patients with a miscarriage or fetal demise were not included in the chart review. Additional information regarding the countries where CBRC was obtained was recorded. Due to the retrospective nature of the study, information about the use of donor gametes, gestational surrogacy, and preimplantation genetic testing could only be obtained if explicitly documented in the patients’ health records. However, none of the electronic health records reviewed indicated the use of donor gametes or gestational carriers. Descriptive statistics were used to summarize baseline characteristics. Continuous variables were compared using the Student’s *t*-test, while categorical variables were analyzed using Fisher’s exact test or the chi-square test (*χ*^*2*^), as appropriate. Statistical significance was defined as *p* < 0.05.

## Results

A retrospective chart review of delivery records at an academic tertiary public hospital was performed. Records were reviewed for one year between February 1, 2023, through January 31, 2024. During this period, there were a total of 4475 patients that delivered at our institute, from which 95 patients were identified to have conceived by IVF, 2.12% of total deliveries. There were 23 patients (24.2%) that sought CBRC to become pregnant with IVF, while 69 patients (72.6%) conceived with IVF in the USA. After the USA, the country most commonly used by patients to conceive was Panama (8.4%), while the remainder of patients were more evenly distributed between Central and South America, including Dominican Republic (2.1%), Brazil (2.1%), and Colombia (2.1%), while for three patients (3.2%), the locations of IVF were unable to be determined from reviewed records, Table 1.

**Table 1:** Country where IVF was performed

Country	*n* (%)
USA	69 (72.6%)
Argentina	1 (1.1%)
Bahamas	1 (1.1%)
Brazil	2 (2.1%)
Colombia	2 (2.1%)
Dominican Republic	2 (2.1%)
Greece	1 (1.1%)
Lebanon	1 (1.1%)
Mexico	2 (2.1%)
Panama	8 (8.4%)
Peru	1 (1.1%)
Unknown	3 (3.2%)
US Virgin Islands	2 (2.1%)

There was no statistically significant difference in maternal characteristics amongst patients who conceived in the USA or outside of the USA. The average age of patients who underwent IVF in the USA was 37.8 years old compared to 40.3 years old for those who underwent IVF internationally. Furthermore, of the patients that became pregnant with IVF in the USA, 42 (60.9%) were nulliparous and 27 (39.1%) were multiparous. A similar distribution was noted in those that were conceived internationally, where 17 (73.9%) were nulliparous while 6 (26.1%) were multiparous. In addition, the indications for IVF were not significantly different in both groups (*p* = 0.90). The main indication was ovulatory dysfunction or diminished ovarian reserve (36.2% in the USA vs 43.5% outside of the USA), followed by male factor (17.4% in the USA vs 17.5% outside the USA), Table 2.

**Table 2:** Patient characteristics

Patient characteristics	Location of IVF		*p*-value
	USA *n* = 69	Outside USA *n* = 23	
Age (yo; mean ± SD)	37.8 ± 5.1	40.3 ± 6.2	0.07
Parity (*n*, %)			0.33
Nulliparous	42 (60.9%)	17 (73.9%)	
Multiparous	27 (39.1%)	6 (26.1%)	
Indication for IVF (*n*, %)			0.90
DOR/ovulatory dysfunction	25 (36.2%)	10 (43.5%)	
Male factor	12 (17.4%)	4 (17.5%)	
Tubal factor	10 (14.5%)	3 (13.0%)	
RPL	7 (10.2%)	3 (13.0%)	
Other	15 (21.7%)	3 (13.0%)	

*IVF*, in vitro fertilization; *DOR*, diminished ovarian reserve; *RPL*, recurrent pregnancy loss

Comparing IVF cycle outcomes performed in the USA to CBRC, the average gestational age at delivery was slightly higher in the US group (37w3d) compared to those outside the USA (37w0d), but this difference was not statistically significant (*p* = 0.41). Cesarean deliveries were predominant in both groups, with 72.5% in the USA and 73.9% outside the USA. Birth plurality showed that singleton deliveries were more common in the US group (88.4%) than outside the USA (73.9%), though the difference did not reach statistical significance (*p* = 0.11). Of note, there was a higher-order multiple gestations consisting of triplets in the CBRC group. Gestational age at delivery categorized as term, late preterm, or preterm revealed that term deliveries were more frequent in the US group (75.4%) compared to the group outside the USA (65.2%), with late preterm and preterm deliveries occurring at higher rates outside the USA (17.4% in the USA vs 21.7% outside of the USA; 7.2% in the USA vs 13.1% outside of the USA, respectively). Average birth weights were slightly higher for the USA group (2926.4 ± 754.2 g) compared to outside the USA (2732.2 ± 834.4 g), though this difference was not statistically significant (*p* = 0.29). Amongst maternal complications, hypertensive disorders were significantly more prevalent outside the USA (69.6%) compared to the USA (37.7%) (*p* = 0.01). Although the remainder of maternal complications did not reach statistical significance, gestational diabetes mellitus (GDM) was more frequent in the US group (13.0%) compared to outside the USA (4.3%), and postpartum hemorrhage (PPH) rates were higher outside the USA (17.4%) than in the USA (5.8%). Neonatal and fetal complications showed similar rates across both groups for conditions such as fetal growth restriction/low birth weight (FGR/LBW), respiratory distress syndrome (RDS), and hyperbilirubinemia, with no statistically significant differences observed, Table 3.

**Table 3:** Delivery outcomes and maternal and neonatal/fetal complications

Delivery and complications	Location of IVF		*p*-value
	USA	Outside USA	
Gestational age at delivery	37w3d	37w0d	0.41
Mode of delivery (*n*, %)			
Vaginal delivery	19 (27.5%)	6 (26.1%)	1.00
Cesarean delivery	50 (72.5%)	17 (73.9%)	
Birth plurality (*n*, %)			0.11
Singleton	61 (88.4%)	17 (73.9%)	
Twin	8 (11.6%)	5 (21.7%)	
Higher order multiple	0	1 (4.3%)	
Gestational age at delivery (*n*, %)			
Term (> / 37.0 wks)	52 (75.4%)	15 (65.2%)	0.42
Late preterm (34.0–36.6 wks)	12 (17.4%)	5 (21.7%)	0.76
Preterm (< 34.0 wks)	5 (7.2%)	3 (13.1%)	0.41
Birth weight, g (mean ± SD)	2926.4 ± 754.2	2732.2 ± 834.4	0.29
Maternal complications (*n*, %)			
Hypertensive disorders	26 (37.7%)	16 (69.6%)	0.01
GDM	9 (13.0%)	1 (4.3%)	0.44
PPH	4 (5.8%)	4 (17.4%)	0.10
Chorioamnionitis/endometritis	5 (7.2%)	2 (8.7%)	1.00
Other	14 (20.3%)	4 (17.4%)	1.00
Neonatal/fetal complications (*n*, %)			
FGR/LBW	7 (9.1%)	5 (14.3%)	0.77
RDS/apnea	11 (14.3%)	5 (14.3%)	0.54
LGA	6 (7.8%)	1 (2.9%)	0.68
Hyperbilirubinemia	9 (11.7%)	3 (8.6%)	1.00
Other	6 (7.8%)	3 (8.6%)	0.69

*IVF*, in vitro fertilization; *GDM*, gestational diabetes; *PPH*, postpartum hemorrhage; *FGR*, fetal growth restriction; *LBW*, low birth weight; *RDS*, respiratory distress syndrome; *LGA*, large for gestational age

## Discussion

This retrospective review of 4475 deliveries at a tertiary hospital found 95 patients conceived using IVF. The incidence of ART among patients delivering at our institution was 2.12%, and this is in agreement with published data regarding the incidence of ART and deliveries in the USA [3]. Of the IVF cases, 24.2% pursued cross-border care, mainly in Panama, while 72.6% underwent IVF in the US maternal characteristics, and IVF indications were similar, though international IVF patients were slightly older (40.3 vs. 37.8 years). While maternal and neonatal outcomes were largely comparable between CBRC and domestic IVF pregnancies, a notably higher incidence of hypertensive disorders was observed in the CBRC group (69.6% vs. 37.7%), reaching statistical significance. Trends toward increased rates of postpartum hemorrhage and multiple gestations were also noted but did not achieve statistical significance. Neonatal complications, including fetal growth restriction and respiratory distress, were similar.

Cross-border reproductive care is increasingly accessed internationally. Between 2006 and 2013, the use of ART cycles performed in the US to non-US residents more than doubled from 1.2 to 2.8% [4], while in Europe, it is estimated that approximately 5% of all fertility care was the product of CBRC in 2010 [5, 6]. However, it is not known how many US residents are leaving the country to access reproductive care due to a lack of an international reporting system. While our study is limited to a single tertiary care academic public hospital, it found that a significantly high number of patients who conceived with ART, 24.2%, accessed CBRC to achieve pregnancy. With such a large number of patients seeking care abroad, it is important to understand their motivations and potential risks associated with such a trend. The reasons driving patients to seek fertility care abroad are multifaceted, intricate, and frequently interconnected.

The cost of fertility care within the USA can be prohibitive to patients seeking care. With the cost of a single IVF cycle including medications averaging $20,000 to $25,000 [7, 8], patients often require more than one cycle to achieve a live birth, and with the lack of insurance coverage in the majority of states [9, 10], the cost of achieving a live birth can be burdensome to patients. Many individuals pursue fertility treatments in countries where ART is more affordable than in their home countries, despite concerns about potentially lower success rates [11]. The average cost of an IVF cycle in the USA is the highest globally [8] and has been a reason cited by patients accessing CBRC [12]. Meanwhile, Latin America accounted for nearly all of the CBRC cycles in our study, except for one cycle in Greece and one in Lebanon. In the most frequently used country for CBRC by our population, Panama, the cost of an IVF cycle is significantly lower at approximately $5600, based on available data from fertility clinics. Other countries accessed by patients in our study have significantly less costly IVF cycles as well, such as $5000 to $8000 in Mexico and the Dominican Republic, from similar data.

Another reason frequently cited by patients for accessing CBRC is for cultural preference and privacy. Having a shared language and culture with a medical provider is a factor important to patients traveling abroad for care [13–15]. Similarly, “return reproductive tourism” describes migrants returning to their home countries for CBRC due to reasons such as familiarity with language and religion, as well as increased trust in the home-country ART services [16, 17]. This phenomenon may explain cases of CBRC in our population in which the county is composed of over 50% immigrants [18]. Conversely, some patients have also reported a lack of support from family and friends during infertility treatment or desire privacy to avoid social stigma [19, 20], prompting them to seek privacy in another country.

While CBRC offers opportunities for individuals to access ART services that may be unavailable or unaffordable in their home countries, it also presents several risks. Although our study did not find a statistically significant difference in the number of multiple pregnancies, the number of multiple pregnancies was more than doubled in patients conceiving through CBRC (11.6% vs 26.1%), and there was also a triplet gestation in this cohort. This increased risk has been confirmed in other publications of patients accessing CBRC [21–23], which brings along associated risks of twin gestations, such as preterm delivery, cesarean delivery, and neonatal intensive care admissions [24]. When accessing CBRC, it may be possible that infertility providers are not adhering to embryo transfer guidelines, such as those from the American Society for Reproductive Medicine in the USA [25]. Furthermore, our study found nearly double the number of preterm deliveries prior to 34w0d (13.1% vs 7.2%) and an increased number of late preterm deliveries, 34w0d to 36w6d (21.7% vs 17.4%) in CBRC pregnancies, a risk that has been previously described [21, 23].

Patients accessing CBRC were of higher age compared to those who conceived in the USA (40.3 vs 37.8 years old). Although our finding was not statistically significant, it is known that conception at advanced maternal age poses greater risks of complications during the pregnancy such as cesarean delivery, hypertensive disorders, preterm birth, and low birth weight [26, 27]. The majority of patients accessing CBRC report undergoing prior treatments in their home countries, often spanning several years. Many also have a history of failed treatment attempts, which motivates them to seek care abroad [13, 28–30]. This prolonged treatment journey may contribute to the older age observed in our study population. Furthermore, knowledge about lower pregnancy rates with advanced age and the resulting need for multiple cycles may prompt patients of older age to seek care outside of the USA where treatment is more affordable.

Our study revealed that patients who conceived with CBRC had a statistically significant greater percentage of hypertensive disorders as well as an increase in other maternal complications, including PPH and chorioamnionitis/endometritis, Table 3. These findings may be explained by the increasing age of patients in the CBRC cohort, the increased number of multiple pregnancies, or reasons specific to more aggressive treatment protocols used internationally where emphasis is placed on cycle outcomes [2]. Furthermore, while there was no significant difference in fetal/neonatal complications, our study found a larger proportion of FGR/LBW (14.3% vs 9.1%) in pregnancies conceived outside the USA, consistent with published data [23, 31]. This finding may be associated with the increase in hypertensive disorders and older age found in patients accessing CBRC.

This study’s primary strength lies in its comprehensive retrospective analysis of 4475 deliveries at a tertiary public hospital, with a specific focus on maternal and neonatal outcomes in CBRC conceptions. By including a diverse patient population from various countries and comparing outcomes with US-based IVF conceptions, the study provides valuable insights into potential risks and complications associated with CBRC. The detailed examination of clinical variables such as gestational age, delivery mode, and maternal and neonatal complications strengthens the study’s reliability. However, the study has limitations that should be addressed in future research. The relatively small sample size of CBRC patients may reduce the statistical power to detect significant differences in outcomes. To expand on this work, future research will include a survey-based study exploring patient motivations and experiences with CBRC, including cultural, economic, and personal factors driving international treatment. Furthermore, information will be obtained regarding the use of donor gametes, gestational surrogacy, preimplantation genetic testing, and fresh or frozen embryo transfer, which could affect the percentage of hypertension among CBRC pregnancies that were observed in our study. Additionally, increasing the sample size by including additional years of delivery data will enhance the statistical power and allow for more robust comparisons, ultimately providing a deeper understanding of CBRC’s global impact on maternal and neonatal health. Finally, given the nature of a retrospective chart review study, it is possible that some IVF deliveries were unintentionally excluded if the patient did not disclose this information. However, we are confident that our sample of IVF deliveries (2.12% of all deliveries at the hospital) is reflective of the population, given that 2.3% of all infants born in the USA in 2021 [3].

## Conclusion

This study primarily investigated the incidence of CBRC among IVF pregnancies and evaluated maternal and neonatal outcomes compared to domestic IVF conceptions. CBRC accounted for 24.2% of IVF pregnancies, highlighting its notable presence within the studied population. While most maternal and neonatal outcomes were similar between the two groups, a statistically significant higher incidence of hypertensive disorders was observed in CBRC pregnancies (69.6% vs. 37.7%, *p* = 0.01). Although other outcomes, including preterm birth, postpartum hemorrhage, and neonatal complications, did not reach statistical significance, trends indicated increased maternal risks among CBRC patients. These findings underscore the need for future research with larger sample sizes to enhance statistical power and confirm these associations. Additionally, survey-based studies exploring patient motivations and experiences with CBRC would provide deeper insights into the complex factors driving international fertility treatment decisions and inform personalized patient counseling and care management.

## Data Availability

The datasets used and analyzed during the current study are available from the corresponding author upon reasonable request.
